# Are Steroids Still Useful in Immunosuppressed Patients With Inflammatory Bowel Disease? A Retrospective, Population-Based Study

**DOI:** 10.3389/fmed.2021.651685

**Published:** 2021-06-25

**Authors:** Beatriz Sicilia, Lara Arias, Gadea Hontoria, Nieves García, Ester Badia, Fernando Gomollón

**Affiliations:** ^1^Hospital Universitario de Burgos, Burgos, Spain; ^2^Facultad de Medicina, Hospital Clínico Universitario, Instituto de Investigación Sanitaria de Aragón, Ciberehd, Zaragoza, Spain

**Keywords:** immunosuppression, corticosteroids, Crohn's disease, ulcerative colitis, inflammatory bowel diseases

## Abstract

**Background:** Effectiveness of corticosteroids in immunosuppressed patients with inflammatory bowel disease (IBD) has not been completely elucidated.

**Aims:** To assess the effectiveness and examine the long-term follow-up of systemic or low-bioavailability oral steroid treatment for moderate flare-ups in patients treated with immunosuppressive drugs.

**Methods:** Immunosuppressed patients with inflammatory bowel disease (IBD) from our population-data registry were analyzed. For statistical analysis, the chi-square test, Mann-Whitney U test, and Kaplan-Meier survival analysis were used as appropriate.

**Results:** A total of 392 patients with IBD and a median of 82 (range, 6–271) months of immunosuppressive (IMM) treatment were identified. The mean follow-up was 87 months (range, 6–239 months). A total of 89 patients (23%) needed at least one steroid course during their follow-up. Average time from IMM to steroid treatment was 26 (range, 6–207) months. In patients with CD, fibrostenotic (B2) and fistulizing (B3) behaviors [*p* = 0.005; odds ratio (OR): 2.284] were risk factors for using steroids after IMM treatment. In patients with UC, no statistically significant variables were identified. Of the 89 patients who received one first steroid course, 49 (55%) stepped up to biological treatment or surgery after a median of 13 months (range, 0–178), 19 (21%) were treated with repeated steroid courses, and 31 (35%) required no further treatment. Patients with CD had a higher risk (*p* = 0.007; OR: 3.529) of receiving biological treatment or surgery than patients with UC. The longer the patients with UC (more months) spent using steroids, the greater the risk of requiring treatment with biological drugs or surgery (*p* = 0.009).

**Conclusion:** A total of 23% of the immunosuppressed patients with IBD received at least one course of steroid treatment. In patients under immunosuppression treated with at least a course of steroids, CD patients were more likely stepped up to biologics and/or surgery than UC patients. In patients with CD, B2/B3 behavior pattern were significant risk factors. After one course of steroids only 35% of immunosuppressed IBD patients remained in remission without needing treatment scalation.

## Introduction

In 1955, Truelove and Witts (1) were the first to demonstrate the efficacy of corticosteroids to induce remission in patients with inflammatory bowel disease (IBD): moderate to severe ulcerative colitis (UC) patients treated with hydrocortisone (100 mg/day) did have a clearly better response than those receiving placebo, with statistically significant differences in hard endpoints as mortality. Until now corticosteroids continue to be the most widely used drugs for the treatment of moderate and severe flare-ups in both Crohn's disease (CD) and UC (2, 3).

The main effects of systemic corticosteroids (prednisone or equivalent) are to induce remission of moderate and severe outbreaks in patients with CD [evidence level (EL) 1] (4) and UC (EL 1) (5), with high quality of evidence (QL) and strong recommendation (6). High doses of prednisone (40 mg/day−1 mg/kg) are usually prescribed. Different systemic drugs require specific doses, and parenteral route is sometimes preferred in severe cases. For low-bioavailability systemic corticosteroids, 9 mg of oral budesonide is the treatment of choice to induce remission in patients with ileal CD, both in mild (EL 2) and moderate (EL 1) flares (4). In patients with UC and mild-to-moderate flare-ups without a response to mesalamine, beclomethasone at a minimum dose of 5 mg/day (EL 2) (5) (low QL, weakly in favor) (6) or multimatrix budesonide at a dose of 9 mg/day (EL 2) can be used (Table 1). Steroid-resistance (primary failure) and steroid-dependence are common and defined in ECCO guidelines (4).

**Table 1:** Level of evidence and indications for use of corticosteroids in patients with IBD.

	**Flare-up**	**Corticosteroid**	**ECCO**	**GRADE**
UC	Mild-moderate	Beclomethasone 5 mg/d	EL 2	QL low RG Weak for
	Mild-moderate	Budesonide MMX 9 mg/d	EL 2	–
	Mild-Moderate	Prednisone 1 mg/kg	EL 1	QL moderate RG Strong for
	Severe	Prednisone 1 mg/kg	EL 1	QL high RG Strong for
CD	Mild ileocecal	Budesonide 9 mg/d	EL 2	–
	Moderate ileocecal	Budesonide 9 mg/d	EL 1	–
	Moderate-severe	Prednisone 1 mg/kg	EL 1	–
	Severe	Prednisone 1 mg/kg	EL 1	–

*QL, Quality of evidence; EL, Evidence level; RG, Recommendation Grade*.

Thiopurine immunosuppressant drugs (azathioprine or mercaptopurine) are effective for patients with corticosteroid-dependence and have helped patients achieve a withdrawal rate from corticosteroids of almost 60% (7). Corticosteroids are frequently used in patients already under immunosuppressive (IMM) treatment for controlling a new flare-up. However, scant data are available on the efficacy of corticosteroid use in this scenario. In fact, main published studies have shown that 40–50% of patients receive corticosteroid treatment and that 20–30% receive immunomodulatory treatment (8–11). A recent Canadian study of 3,312 patients with IBD in clinical practice between 1994 and 2014 (12) showed that corticosteroids were prescribed in the first year of IMM treatment for 20% of patients with CD and up to 40% of patients with UC. Steroids have significant toxicity, especially in the long-term. Data on their effectiveness in daily clinic could help for designing new protocols of use.

We do present our clinical experience with the use of steroids in a retrospective study of all the patients from our IBD Unit.

## Aims

Our first aim was to evaluate the effectiveness of systemic oral (prednisone) and low-bioavailability (beclomethasone/budesonide) corticosteroids to induce remission of moderate IBD flare-ups in patients with at least 6 months of IMM treatment (azathioprine, mercaptopurine, or methotrexate). Our second aim was to assess the long-term effectiveness of this treatment in avoiding the need for biological drugs and/or surgery and analyzing the predictors of response in this specific scenario.

## Methodology

We used a population database to identify patients diagnosed with IBD (both UC and CD) between 1966 and 2016 from a reference area of 176,208 inhabitants [Spanish Statistical Office (INE) 2016]; we included only patients who had at least 6 months of IMM treatment in our study.

We performed a descriptive analysis and collected the following variables:

- Type of IBD (UC/CD)
- Year of diagnosis
- Location of IBD according to the Montreal classification for UC and CD:
  - Extensive UC (E3)/left-sided (E2)/ulcerative proctitis (E1)
  - Ileal CD (L1)/colonic (L2)/ileocolonic (L3) with/without isolated upper disease (L4)
  - Presence of perianal disease
- Behavior pattern according to the Montreal classification for CD:
  - Inflammatory pattern (B1)/fibrostenotic (B2)/penetrating (B3)
- Appendectomy
- Smoking habit at diagnosis:
  - Smoker: Smoker at the start of IMM and current treatment
  - Former smoker: At least 6 months without smoking
  - Non-smoker: Never smoked or >10 years without smoking
- Previous surgery
- Use of corticosteroids at diagnosis
- IMM treatment
  - Type of IMM treatment: azatioprine, mercaptopurine, methotrexate
  - IMM treatment time until the use of corticosteroids and until the end of follow-up
- Treatment with biological drugs prior to immunosuppression

During the follow-up, we recorded the following:

- Treatment with systemic oral or low-bioavailability corticosteroids, dosage, and time
- The efficacy of treatment with corticosteroids, which was defined as not requiring further cycles of corticosteroids, escalation of treatment, or surgery
- The amount of time in which no rescue therapy was necessary after the use of corticosteroids
- Any need for biologic drugs, surgery, or further corticosteroid cycles.

Activity of the disease was defined clinically first with physician-based subjective evaluation and then Harvey-Bradshaw index in CD (<4 points defined as clinical remission) and Truelove-Wits index in UC (remission if <3 bowel movements/day and no rectal bleeding). The goal of treatment was clinical remission, and steroids prescribed to obtain remission if clinical activity present. Protocol for steroids in our hospital followed textbook and GETECCU (www.geteccu.org) recommendations in the first years (1966–2006) and ECCO guidelines from 2006 to 2016. In brief, moderate to severe CD or UC were treated with prednisone, with 1 mg/kg/day starting dose, and tapering from week 4 (usually reducing 10 mg/day every week). Mild to moderate ileitis was treated with budesonide (9 mg/day, tapering from week 4). Mild to moderate cases of UC were treated with oral beclomethasone (5–10 mg/day; 1–2 months course).

Patients were treated at the discretion of the responsible physician. In most cases IBD patients were under the care of the same expert gastroenterologist from 1966 to 2010. After 2010 the team responsible for IBD patients has remained constant, following ECCO guidelines as gold-standard. Biologics were available from several months (<12) after EMA approval. Azathioprine was given at 2.5–3 mg/kg/day in one dose, and mercaptopurine at 1.5 mg/kg/day. In both cases doses were adjusted if needed by frequent (every 3–6 months) blood tests follow-up, but no metabolites determination was available.

For statistical analysis, all data were processed using the IBM SPSS 19 statistical software with a confidence interval of 95%. The data had been previously collected and processed using Microsoft Office Excel 2010. Descriptive analysis of the sample was performed and showed the means (standard deviation), medians (interquartile range), and frequency (percentage) according to the characteristics and distributions of variables. Differences between variables were evaluated using the chi-square (Fisher) test for qualitative variables and Student's *t*-test, provided it verified the conditions of use; otherwise, the corresponding non-parametric tests were used, Mann-Whitney U test or Kruskal-Wallis, if one of the variables was quantitative.

Finally, survival analysis was performed using the Kaplan-Meier curve and comparing different survival curves according to the diagnosis of UC or CD using the log-rank hypothesis test, which tests the null hypothesis that the two groups compared have the same survival curves.

## Results

We identified 904 patients with IBD in our population database from 1966 to 2016 and selected 392 (43.3%) who had at least 6 months of IMM treatment, with a mean duration of 82 months (6–271). Of these 392 patients, 260 were diagnosed with CD (66%) and 132 were diagnosed with UC (34%). We describe the demographic and clinical characteristics of the patients in Table 2.

**Table 2:** Baseline patient demographics and disease characteristics.

**IMM Treatment >6 Months**	**CD 260 (66%)**	**UC 132 (34%)**	**Total 392 (100%)**
Gender (male)	136 (52%)	71 (54%)	207 (53%)
Location	46% L1/18% L2/36% L3/ 17% L4	52% E3/45% E2/3% E1	
Behavior	57% B1/43% B2-3 25% perianal disease		
Appendectomy	91 (77%)	6 (5%)	97 (25%)
Extra-intestinal manifestations	49 (19%)	23 (17%)	72 (19%)
Smoke habit	Smoker 84 (32%)	Smoker 17 (13%)	Smoker 101 (26%)
	Former smoker 50 (19%)	Former smoker 12 (9%)	Former smoker 62 (16%)
	Non-smoker 126 (48%)	Non-smoker 103 (78%)	Non-smoker 229 (58%)
Steroids at Dg	200 (77%)	104 (79%)	304 (78%)
Biological before IMM treatment	4 (2%)	0 (0%)	4 (2%)
Surgery before IMM treatment	62 (24%)	2 (16%)	64 (16%)
Steroids post IMM treatment	Classical 38 (63%)	Classical 18 (62%)	Classical 56 (65%)
	Low-bioavailability 22 (37%)	Low-bioavailability 11 (38%)	Low-bioavailability 33 (37%)
Surgery follow-up	13 (5%)	1 (0.8%)	14 (3.5%)
Biologic follow-up	27 (10%)	9 (7%)	35 (9%)

A total of 89 patients (23%) were treated with at least one course of oral corticosteroids during their follow-up, with an average duration of 4 months (1–168 months). Of these 89 patients, 63% received treatment with systemic corticosteroids and 37% with low-bioavailability oral corticosteroids; a total of 29 patients (33%) had UC, and 60 patients (67%) had CD (*p* = 0.805).

**Figure 1:**
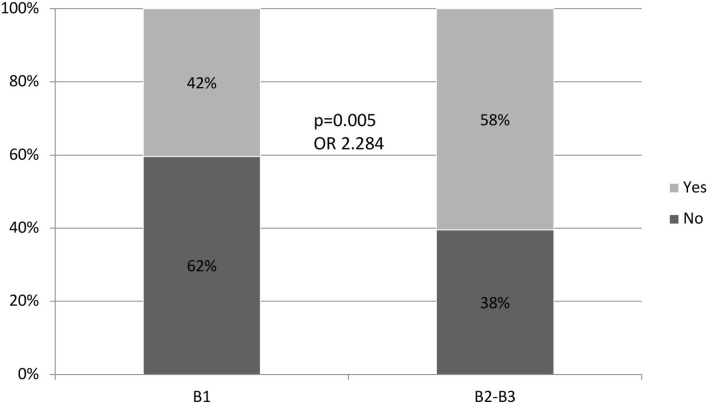
Relation between the pattern of behavior and the need for treatment with corticosteroids.

A comparison of the variables associated with the need for treatment with corticosteroids (Table 3) showed no differences with regard to sex, age, location of the disease, perianal disease, appendectomy, extra-intestinal manifestations, smoking habits, previous use of corticosteroids, and previous surgery. Fibrostenotic (B2) and fistulizing (B3) patterns of CD (*p* = 0.005) behaved as a statistically significant variable. A patient with a fibrostenotic or fistulizing pattern of CD (B2-B3) was twice as likely to take corticosteroids while on IMM treatment than a patient with an inflammatory pattern of CD (B-1) [odds ratio (OR): 2.284] (Figure 1). A total of 4 patients (1%) with CD required treatment with a biological drug prior to starting their IMM treatment (a top-down strategy), and this was also associated with the need for taking corticosteroids during evolution (*p* = 0.038).

**Table 3:** Variables associated with the need for treatment with corticosteroids in patients who have received at least 6 months of immunosuppressant treatment.

**Variables**		**Post-IMM treatment corticosteroids**		
		**No (*n* = 303)**	**Yes (*n* = 89)**	***p***
Sex	Male	160 (53%)	47 (53%)	1.000
	Female	143 (47%)	42 (47%)	
Age		47.6	49.66	0.242
Corticosteroids at diagnosis	No	72 (24%)	14 (16%)	0.102
	Yes	229 (76%)	75 (84%)	
Current diagnosis	UC	103 (34%)	29 (33%)	0.805
	CD	200 (66%)	60 (67%)	
UC location	E3	49 (48%)	20 (69%)	0.097
	E2	50 (49%)	9 (31%)	
	E1	4 (4%)	0 (0%)	
CD location	L3	41 (21%)	6 (10%)	0.105
	L2	67 (34%)	27 (45%)	
	L1	92 (46%)	27 (45%)	
L4	No	170 (85%)	46 (77%)	0.131
	Yes	30 (15%)	14 (23%)	
**Pattern**	**B1**	**76 (38%)**	**35 (58%)**	**0.005** **OR: 2.284**
	**B2/B3**	**124 (62%)**	**25 (42%)**	
Perianal disease	No	258 (85%)	69 (78%)	0.089
	Yes	45 (15%)	20 (22%)	
Extra-intestinal manifestations	No	244 (81%)	72 (82%)	0.918
	Yes	56 (15%)	16 (18%)	
**Biological drugs prior to IMM treatment**	**No**	**302 %)**	**86 (97%)**	**0.038**
	**Yes**	**1 (0%)**	**3 (3%)**	
Surgery prior to IMM treatment	No	257 (85%)	71 (80%)	0.258
	Yes	46 (15%)	18 (20%)	
Smoker	No	169 (56%)	60 (67%)	0.147
	Yes	83 (27%)	18 (20%)	
	Former smoker	51 (17%)	11 (12%)	
Appendectomy	No	225 (76%)	59 (69%)	0.151
	Yes	70 (24%)	27 (31%)	

*The bold values highlight the variables which are statistically significative*.

A total of 49 patients (55%), who were on IMM treatment and treated with corticosteroids for a moderate flare-up of their disease, required biological treatment or surgery during their average follow-up period of 40 months (1–178 months); a total of 19 patients (21%) required more than 1 cycle of corticosteroids. The mean length of time from taking the immunosuppressant drug to the use of corticosteroids was 26 months (6–207). There is no predictive variable of corticosteroid efficacy in patients with CD, but there is a directly proportional association between the time of corticosteroid use and the need for treatment with biological drugs or surgery in the follow-up of patients with UC (Tables 4, 5).

**Table 4:** Requirement for rescue therapy with biological drugs/surgery in immunosuppressed patients with UC who have needed treatment with corticosteroids.

**Corticosteroids after IMM treatment (*****n*** **=** **29)**		**Biological drugs/surgery UC**				
		**No (*****n*** **=** **19)**		**Yes (*****n*** **=** **10)**		
		**Median/n**	**Range/%**	**Median/n**	**Range/%**	***p***
Age (mean/deviation)		53.63	±11.63	44.2	±12.67	0.054
Sex	Male	11	58%	5	50%	0.714
	Female	8	42%	5	50%	
Corticosteroids at diagnosis	No	1	5%	3	30%	0.105
	Yes	18	95%	7	70%	
Location	E3	13	68%	7	70%	1.000
	E2	6	32%	3	30%	
	E1	0	0%	0	0%	
Extra-intestinal manifestations	No	17	89%	8	80%	0.592
	Yes	2	11%	2	20%	
Smoker	No	18	95%	7	70%	–
	Yes	0	0	1	10%	
	Former smoker	1	5%	2	20%	
Appendectomy	No	19	100%	8	80%	0.111
	Yes	0	0%	2	20%	
Corticosteroids	Low-bioavailability	9	47%	2	20%	0.234
	Standard	10	53%	8	80%	
**Months on corticosteroids**		**2**	**(2–7)**	**5**	**(3–44)**	**0.004**

*The bold values highlight the variables which are statistically significative*.

**Table 5:** Requirement for rescue therapy with biological drugs/surgery in immunosuppressed patients with CD who have needed treatment with corticosteroids.

**Corticosteroids after IMM treatment (*****n*** **=** **60)**		**Biological drugs/surgery CD**				
		**No (*****n*** **=** **21)**		**Yes (*****n*** **=** **39)**		***p***
		**Median/n**	**Range/%**	**Median/n**	**Range/%**	
Age (mean/deviation)		48.71	±15.60	49.64	±13.90	0.814
Sex	Male	10	48%	21	54%	0.645
	Female	11	52%	18	46%	
Corticosteroids at diagnosis	No	5	24%	5	13%	0.298
	Yes	16	76%	34	87%	
Location	Colonic	4	19%	2	5%	–
	Ileal	9	43%	18	46%	
	Ileocolonic	8	38%	19	49%	
L4	No	18	86%	28	72%	0.340
	Yes	3	14%	11	28%	
Pattern	B2/B3	12	57%	23	59%	0.891
	B1	9	43%	16	41%	
Extra-intestinal manifestations	No	15	75%	32	82%	0.524
	Yes	5	25%	7	18%	
Perianal disease	No	14	67%	26	67%	1.000
	Yes	7	33%	13	33%	
Smoker	No	12	57%	23	59%	
	Yes	5	24%	12	31%	
	Former smoker	4	19%	4	10%	
Appendectomy	No	11	58%	21	55%	0.850
	Yes	8	42%	17	45%	
Corticosteroids	Low-bioavailability	10	48%	12	31%	0.196
	Standard	11	52%	27	69%	
Months on corticosteroids		4	(2–30)	4	(1–168)	0.511

A single cycle of corticosteroids was effective in 31 patients (35%), and no other type of treatment was needed in these patients during their follow-up period, such as further cycles of corticosteroids, biologics, or surgery.

**Figure 2:**
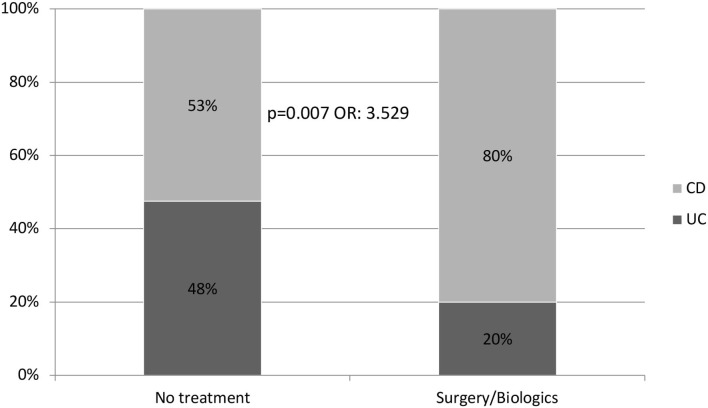
Probability of receiving biological treatment and/or surgery during follow-up after receiving steroids (89 patients).

**Figure 3:**
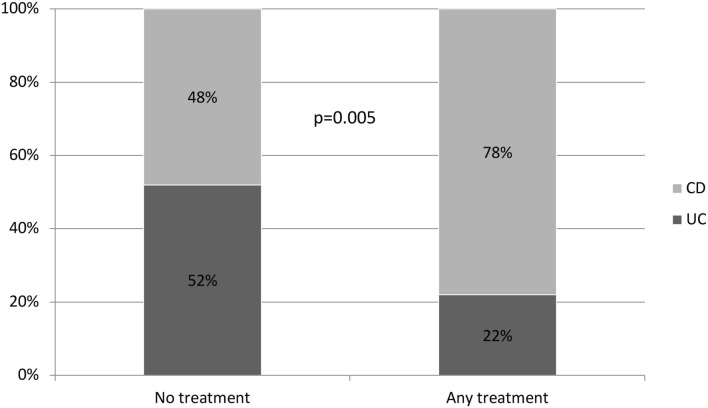
Probability of NO treatment at all (No biological drug, surgery, or corticosteroids).

A comparison of the variables associated with the need for biological drugs or surgery showed a higher risk of treatment with a biological drug or surgery after initial corticosteroid treatment in patients with CD than in patients with UC (*p* = 0.007; OR: 3.529) (Figure 2). Of the 89 patients who required treatment with corticosteroids, 10 with UC (20%) and 39 with CD (80%) needed rescue therapy throughout their follow-up. This difference was maintained when analyzing the probability of not requiring any type of treatment, including repeat cycles of corticosteroids (*p* = 0.005) (Figure 3).

**Figure 4:**
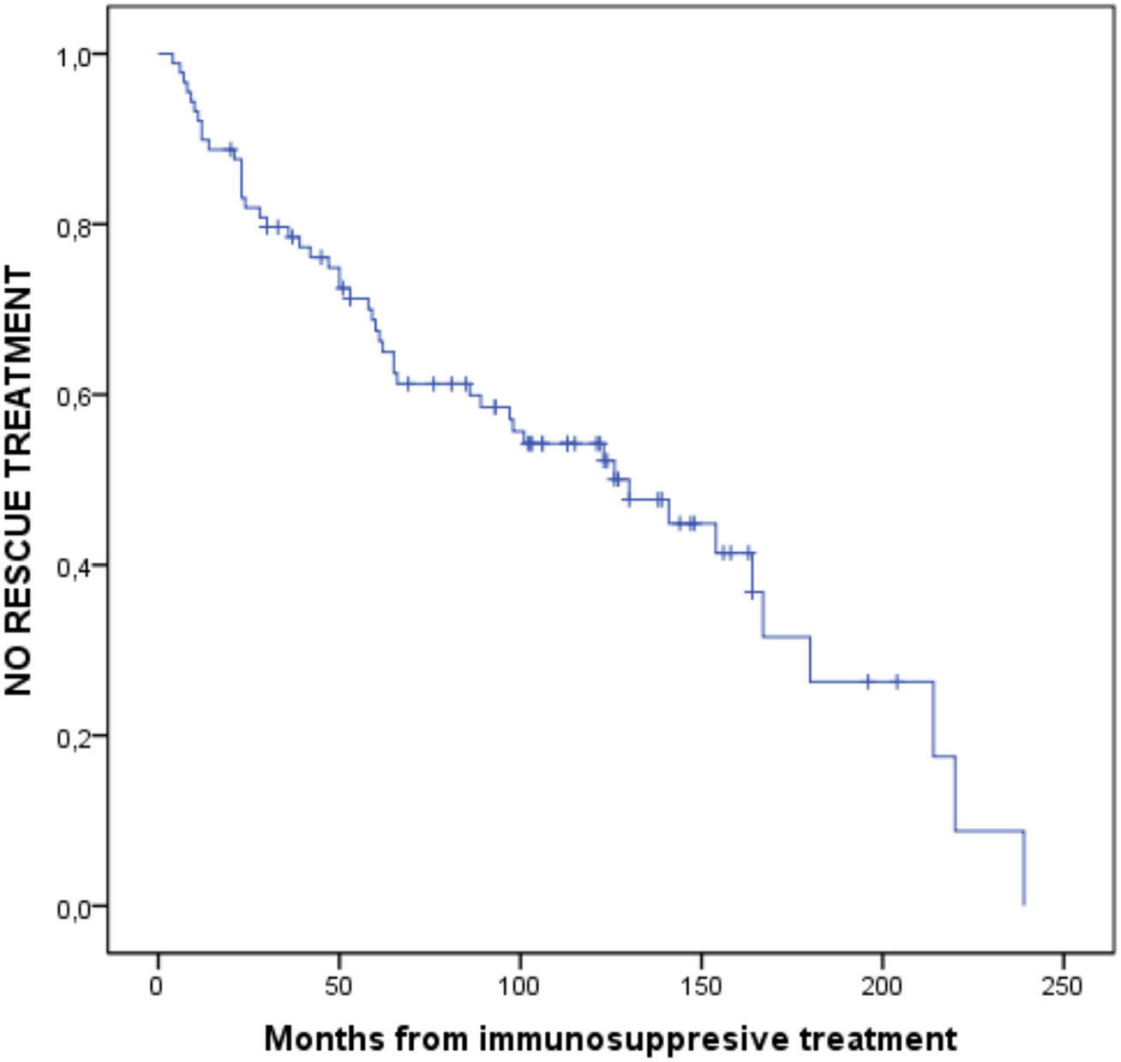
Probability of NO rescue therapy from IMM treatment to the end of follow-up.

**Figure 5:**
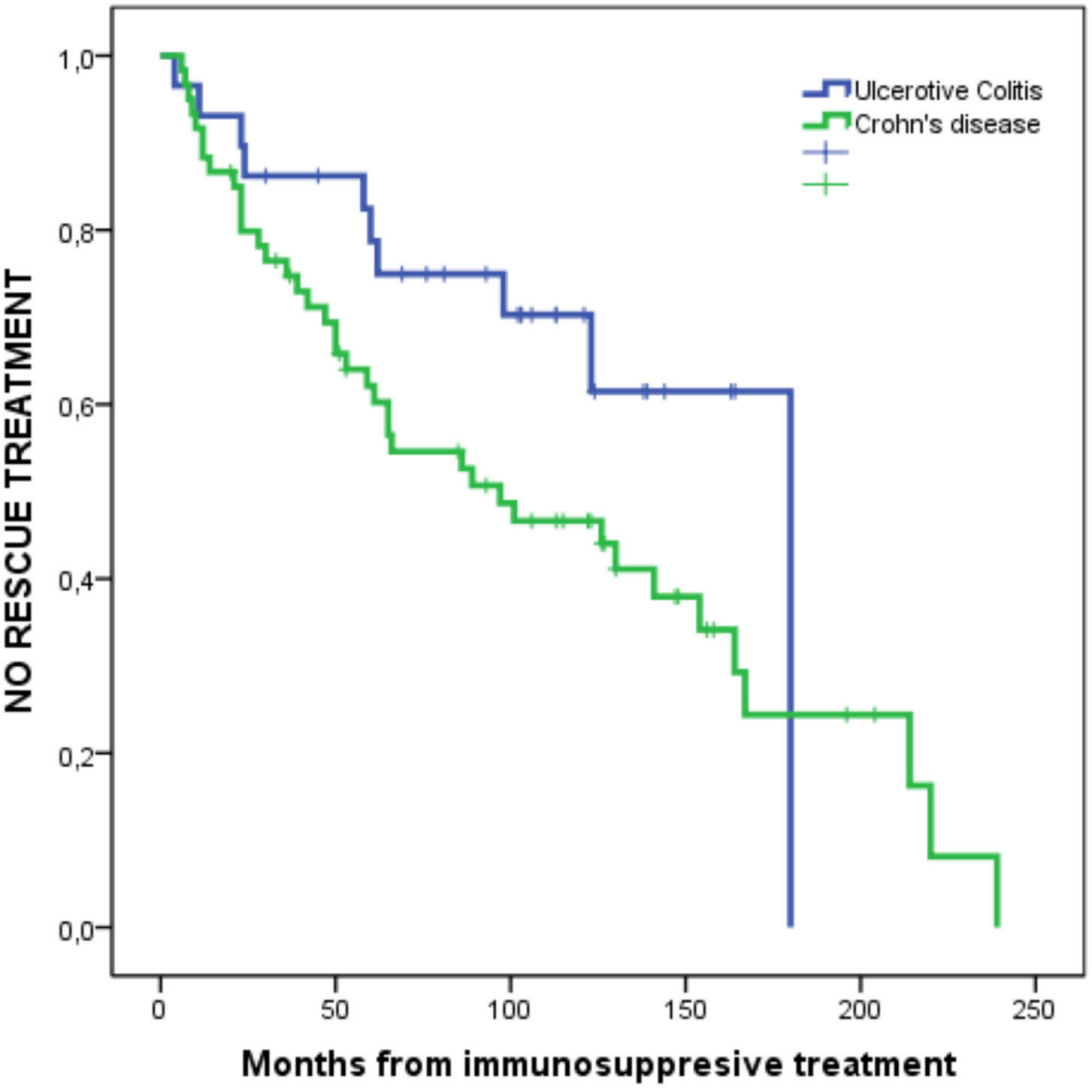
Probability of NO rescue therapy from IMM treatment to the end of follow-up in UC and CD. *P* = 0.078.

In the survival analysis (Figure 4), we observed a 50% chance of not requiring biological drugs or surgery at 130 months. When comparing survival between patients with CD and those with UC (Figure 5), we observed a clear separation between both curves, but they crossed at certain periods (*p* = 0.078); however, survival without salvage therapy was higher in patients with UC. The 50% chance of not receiving any type of treatment after receiving corticosteroids lasted 83 months longer (range 97–180) in patients with UC compared to those with CD (Table 6). We observed a 75% probability of not needing any additional treatment for 62 months in patients with UC and for 36 months in patients with CD (Table 7).

**Table 6:** Means and medians of survival times.

**Current diagnosis**	**Mean^a^** **(months)**			**Median (months)**		
	**Estimate**	**95% confidence interval**		**Estimate**	**95% confidence interval**	
		**Lower limit**	**Upper limit**	**Lower limit**	**Lower limit**	**Upper limit**
Ulcerative colitis	134,96	109,21	160,73	180,00	–	–
Crohn's disease	111,10	88,47	133,75	97,00	32,12	161,87
Total	122,27	102,87	141,68	130,00	82,79	177,21

a *The estimate is restricted to the longest survival time if this was recorded*.

**Table 7:** Percentils.

**Current diagnosis**	**25.0%**	**50.0%**	**75.0%**
	**Estimate (months)**	**Estimate (months)**	**Estimate (months)**
Ulcerative colitis	180	180	62
Crohn's disease	167	97	36
Total	214	130	47

## Discussion

The use of corticosteroids in our patients who had responded to IMM treatment reached 55%, with no differences between patients with UC and patients with CD, and 21% required more than one course of corticosteroids. We believe this percentage is likely higher than in current clinical practice, because our review was retrospective and included years when biological drugs were not yet in use. The pivotal published studies (8–11) report corticosteroid treatment in 40–50% of patients enrolled in clinical trials, and ~30% did not respond to IMM treatment. The only randomized clinical trial that reported this associated data was the GEMINI 1 (9), where 17–21% of the patients included in the different treatment arms were receiving IMM treatment and taking corticosteroids concurrently.

Reinforcing the results of other published cohorts (13), the results of our study show that the fibrostenotic and fistulizing patterns in CD are statistically significant factors associated with the need for corticosteroid treatment in immunosuppressed patients; likely because this reflects a greater severity in the clinical evolution of this behavior pattern and has been reflected in follow-up, epidemiological-incident cohort studies (14). However, compared to the inflammatory pattern, this pattern does not predict the efficacy of this strategy, which is defined as the subsequent requirement of treatment with biological drugs or surgery.

Perianal disease, young age at diagnosis, and the need for corticosteroids appear to be risk factors in patients with CD; therefore, we attempted to identify the criteria and clinical factors associated with a more aggressive course to coincide with the emergence of more effective therapies (15, 16). In our cohort, this variable was not associated with a subsequent need for corticosteroids once the patient is already on immunosuppressive therapy. In addition, the use of corticosteroids at diagnosis did not predict the need for subsequent rescue therapies once these corticosteroids were used in our patients. However, once the patient is receiving IMM treatment, as is the case with our cohort, the use of corticosteroids should be carefully evaluated. At no instance should more than 1 cycle of corticosteroid (preferably of low-bioavailability) be used if other therapeutic options that are more effective in the long-term have not been optimized, such as the use of biological drugs and surgery.

The overall efficacy of corticosteroid use in already-immunosuppressed patients with UC or CD, which was defined in our study as having no need for any other type of treatment throughout the follow-up, was only 35%; therefore, 65% of patients receiving corticosteroids will require a new cycle of treatment with corticosteroids, biological drugs, or surgery.

The efficacy of this course of corticosteroids for the long-term in our cohort was significantly different between patients with UC and CD. A patient with CD who needs corticosteroids is 3.5 times more likely to need rescue therapy with biological drugs or surgery than a patient with UC (*p* = 0.007, OR: 3.529). In addition, patients with CD also have a significantly higher risk of requiring more than one cycle of corticosteroids compared to patients with UC.

The role of low-bioavailability corticosteroids in immunosuppressed patients has not been studied. In our cohort, treatment with low-bioavailability corticosteroids in patients with either UC or CD did not predict the use of subsequent salvage therapy when compared to treatment with systemic corticosteroids. Thus, the severity of the flare-up is inadequate to identify the subgroup of patients in which a corticosteroid cycle will be effective. Once we used corticosteroids, we found no differences in the different variables when trying to predict which patients would benefit from this strategy.

Our results indicate that the patients who will need to use corticosteroids the most in the immunosuppressed situation (EC B2-B3) will also be the ones less responsive in the long-term and require early biological treatment or rescue surgery. Therefore, the strategy of using corticosteroids in patients already on IMM treatment may be used most effectively in patients with UC whose flare-up is controlled with low-bioavailability corticosteroids (fewer side-effects). Moreover, the durability of the effect of corticosteroids is greater in these patients with UC than patients with CD.

There are no many studies in the literature that analyze the efficacy of corticosteroids in patients with IMM treatment with which to compare our results; therefore, we believe that data from other larger population cohorts or prospective scenarios are needed to confirm our results. Our work is the first in the literature to show efficacy data and risk factors that are predictive of favorable clinical progress in this specific scenario. We acknowledge the that there are limitations in our study. In fact, there have been many changes in practice during the 50 years observation period, and the retrospective design limits the analysis. However, we think these data add information to our knowledge of natural history of IBD under immunosuppressive treatment.

## Conclusions

More than half of our patients who were in established IMM treatment required treatment with systemic or low-bioavailability corticosteroids throughout their subsequent follow-up. The patients' B2-B3 CD pattern and previous use of biological drugs were the only associated risk factors.

This drug strategy was clearly effective only in 35% (1/3) of the patients treated with corticosteroids; the remaining patients needed further courses of corticosteroids, biological drugs, or surgery. It seems that most flare-ups in IBD patients under immunosuppressants lead finally to biological and/or surgical therapies, and as steroids have significant toxicity, a different strategy could be more adequate, such as directly switching to biologics or considering surgery, depending on individual factors.

Patients with CD who require corticosteroids in this specific scenario are 3.5 times more likely to need rescue therapy with biological drugs or surgery than patients with UC; this is reflected in survival curves during follow-up, where the 50% probability of not receiving any rescue therapy is 83 (180–97) months longer in patients with UC than patients with CD.

## Data Availability Statement

The raw data supporting the conclusions of this article will be made available by the authors, without undue reservation.

## Ethics Statement

The studies involving human participants were reviewed and approved by Ethics Committee Hospital Universitario de Burgos. The patients/participants provided their written informed consent to participate in this study.

## Author Contributions

All authors listed have made a substantial, direct and intellectual contribution to the work, and approved it for publication.

## Conflict of Interest

The authors declare that the research was conducted in the absence of any commercial or financial relationships that could be construed as a potential conflict of interest.

## Abbreviations

CD: Crohn's disease
CI: confidence interval
EL: evidence level
IBD: inflammatory bowel disease
IMM: immunosuppressive
OR: odds ratio
UC: Ulcerative colitis.
